# Knee Cartilage Quantification: Performance of Low-Field MR in Detecting Low Grades of Chondropathy

**DOI:** 10.3390/jimaging11110401

**Published:** 2025-11-08

**Authors:** Francesco Pucciarelli, Antonio Marino, Maria Carla Faugno, Giuseppe Argento, Edoardo Monaco, Andrea Redler, Nicola Maffulli, Pierfrancesco Orlandi, Marta Zerunian, Domenico De Santis, Michela Polici, Damiano Caruso, Marco Francone, Andrea Laghi

**Affiliations:** 1 Radiology Unit, Department of Surgical and Medical Sciences and Translational Medicine, Sapienza University of Rome, Sant’Andrea Hospital, 00189 Rome, Italymariacarla.faugno@uniroma1.it (M.C.F.);; 2 Department of Orthopaedic Surgery, Sapienza University of Rome, Sant’Andrea Hospital, 00189 Rome, Italy; 3 Department of Biomedical Sciences, Humanitas University, IRCCS Humanitas Research Hospital, 20100 Rozzano, Italy; 4 Department of Diagnostic Imaging, IRCCS Humanitas Research Hospital, 20100 Rozzano, Italy

**Keywords:** T2-mapping, T2-relaxation time, low-field MRI, chondropathy, knee

## Abstract

This study aimed to evaluate the diagnostic accuracy of T2 mapping on low-field (0.31 T) MRI for detecting low-grade knee chondropathy, using arthroscopy as the reference standard. Fifty-two patients (mean age 48.1 ± 17.2 years) undergoing arthroscopy for anterior cruciate ligament or meniscal tears were prospectively enrolled, excluding those with previous surgery, infection, or high-grade chondropathy (Outerbridge III–IV). MRI was performed with a 0.31 T scanner using a 3D SHARC sequence, and T2 relaxometric maps were generated for 14 cartilage regions per knee according to the WORMS classification. Arthroscopy, performed within one month by two blinded surgeons, served as the gold standard. A total of 728 regions were analyzed. T2 mapping differentiated healthy cartilage (grade 0) from early chondropathy (grades I–II) with an optimal cut-off of 45 ms and moderate discriminative accuracy (AUC = 0.714 for Reader 1 and 0.709 for Reader 2). Agreement with arthroscopy was good (κ = 0.731), with excellent intra-reader (ICC = 0.998) and good inter-reader reproducibility (ICC = 0.753). Most degenerative changes were located at the femoral condyles (59%). Low-field T2 mapping showed good diagnostic performance and reproducibility in detecting early cartilage degeneration, supporting its potential as a cost-effective and accessible quantitative biomarker for the assessment of cartilage integrity in clinical practice.

## 1. Introduction

Osteoarthritis (OA) is a type of arthritis that affects mostly hips, knees, feet, and hand joints and causes pain and loss of function. OA affects an estimated 32 million people in the US and more than 240 million people worldwide [1]. OA is estimated to cost USD 303 billion annually in medical costs and lost earnings [2]. Joint malalignment, obesity, trauma, meniscal deformity, or anterior cruciate ligament (ACL) tears are the most common causes of OA [3]. Articular cartilage is composed of water (between 65 and 80%) and the percentage of dry weight is approximately 60% collagen and 12% sulfated proteoglycan (PG) [3]: PG loss and degeneration of the collagen network are the initial changes in the pathologic degeneration of cartilage, initiating increased mobility of water and a consequent increase in water content [4]. OA is diagnosed clinically and plain radiography, magnetic resonance imaging (MRI), and computed tomography (CT) are very useful in the evaluation of articular cartilage [5]. However, due to the limits in morphologic cartilage imaging sequences, there is limited sensitivity for early cartilage degeneration [5]. In particular, T2 mapping sequences are available on high-field MR systems (1.5 T and 3.0 T) and T2 relaxation time could allow the detection of the early stages of OA changes such as decreased PG content, increased free water content, as well as more disordered collagen fibers [6]. Low-field MRI scanners (ranging from 0.1 T to 0.55 T) represent an alternative to high-field MRI in musculoskeletal imaging due to increased flexibility and good quality of results [7]. Potential advantages of low-field MRI scanners include lower costs, safety, and the possibility of making flexible bore configurations. Disadvantages include lower signal, decreased field of view, increased scanning time, less chemical shift, and less benefit from gadolinium [8]. To balance these factors, lower-field approaches are increasingly tailored to specific clinical questions and contexts. This diagnostic accuracy study aimed to evaluate the performance of T2 mapping using low-field MRI compared to arthroscopy in assessing knee cartilage T2 relaxation time and detecting early chondropathy.

## 2. Materials and Methods

This prospective study was IRB approved and informed consent was obtained by all participants. The Ethical Committee approved the project (Prot. n. 363 SA/2021–Rif. CE6587/2021).

### 2.1. Study Population

This is a prospective study conducted at Sant’Andrea Hospital, Sapienza University of Rome, enrolling patients scheduled for arthroscopy due to anterior cruciate ligament (ACL) or meniscal tears. Participants younger than 18 or older than 75 years, with a history of infectious joint disease, or who had undergone previous knee surgery were excluded. Patients presenting with high-grade chondropathy (grade III or IV) were also excluded.

### 2.2. MRI Scanner and Acquisition Protocol

MRI examination was performed with a 0.31 T low-field MR scanner (O-scan, Esaote, Genoa, Italy) using the sequence “3D SHARC” designed for the evaluation of cartilage T2 relaxation time [9]. Standard sequences were previously acquired. After consenting, the patients underwent MRI examination. The procedure involved the patient lying on a dedicated seat, inserting the knee to be examined into the scanner equipped with a dedicated coil, and placing the other leg outside the scanner on a specific support. A sagittal acquisition was performed.

The 3D SHARC (Steady-state High-resolution Acquisition with Rephased Contrast) sequence used in this study is a three-dimensional unbalanced steady-state free precession (SSFP) sequence developed for low-field MRI systems. This technique introduces an additional phase-encoding gradient along the slice-selection direction, enabling full 3D coverage. It is characterized by the use of an unbalanced readout gradient, which separates the Free Induction Decay (FID) and echo components within the same repetition period, allowing for the simultaneous acquisition of two signals with distinct contrast behaviors. These two echoes are subsequently combined on a pixel-by-pixel basis with appropriate weighting to generate a single image with improved signal-to-noise ratio (SNR) and enhanced contrast between articular cartilage and joint fluid. On other MRI platforms, the 3D SHARC sequence can be considered analogous to the DESS (dual-echo steady state) sequence by Siemens [10] or the MENSA sequence by GE [11], with the main difference being that SHARC does not perform fat suppression. In our implementation, the 3D SHARC sequence was acquired using the following parameters: TR = 20 ms, TE = 10 ms, flip angle = 30°, voxel size = 0.78 × 0.78 × 0.78 mm^3^, matrix = 172 × 172, pixel bandwidth = 181.7 Hz/pixel, and number of slices = 92. Two volumetric SHARC acquisitions with different flip angles were obtained within the same protocol and combined to generate relaxometric maps (“T1 map”) for each reconstructed slice. Compared to high-field FSE-based protocols, this unbalanced SSFP approach enables visualization of subtle early cartilage alterations at the voxel level, compensating for the lower intrinsic SNR and spatial resolution typical of low-field systems.

### 2.3. Image Analysis

Image analysis was manually performed by two radiologists (FP and DDS, with 5 and 7 years of experience in MRI, respectively) independently with a dedicated experimental software specifically set on MatLab R2025a (The MathWorks Inc., Natick, MA, USA): a color map superimposed to the cartilage reflected different T2 relaxation times. Once the sequence was acquired, a map was reconstructed. Subsequently, the data obtained was transferred from the console to the analysis workstation using a mobile support. Each knee was divided into 14 regions, according to Whole-Organ Magnetic Resonance Imaging Score (WORMS) [12] classification, and a single free-hand region of interest (ROI) was manually drawn in each region. T2 relaxation times were averaged per ROI (expressed as milliseconds), obtaining a single mean value for each of the 14 regions of interest.

### 2.4. Arthroscopy

Arthroscopy was performed by two orthopedic surgeons with 15 and 10 years of experience within a month after MRI examination, blinded of T2 mapping results. The grade of chondropathy, according to Outerbridge [13] classification, was calculated. This classification is based on direct visualization of the joint, either arthroscopic or open, and classifies macroscopic joint cartilage abnormalities into four grades: the system assigns a grade of 0 through IV to the chondral area of interest [13].

### 2.5. Statistical Analysis

Statistical analysis was performed using MedCalc (MedCalc Software, version15, Ostend, Belgium). Continuous variables were expressed as mean ± standard deviation (SD) or as interquartile range (IQR), as appropriate. The diagnostic performance of T2 mapping in differentiating healthy cartilage (grade 0) from pathological cartilage (grades I–II) was assessed using receiver operating characteristic (ROC) curve analysis. The optimal cut-off value was determined by maximizing the Youden index, and the area under the curve (AUC) was calculated to evaluate discriminative accuracy. Agreement between T2 mapping and arthroscopic grading (used as the reference standard) was assessed using the weighted Cohen’s κ coefficient. Intra-reader and inter-reader reproducibility were evaluated using the intraclass correlation coefficient (ICC, two-way random-effects model, single measures, absolute agreement). ICC values were interpreted as follows: <0.50 poor, 0.50–0.75 moderate, 0.75–0.90 good, and >0.90 excellent reliability. A *p*-value < 0.05 was considered statistically significant.

## 3. Results

### 3.1. Study Population

From an initial population of 83 patients, 52 were included in the study (32 males and 20 females). The participants had a mean age of 48.05 ± 17.18 years (range: 18–71) (Table 1).

**Figure 1:**
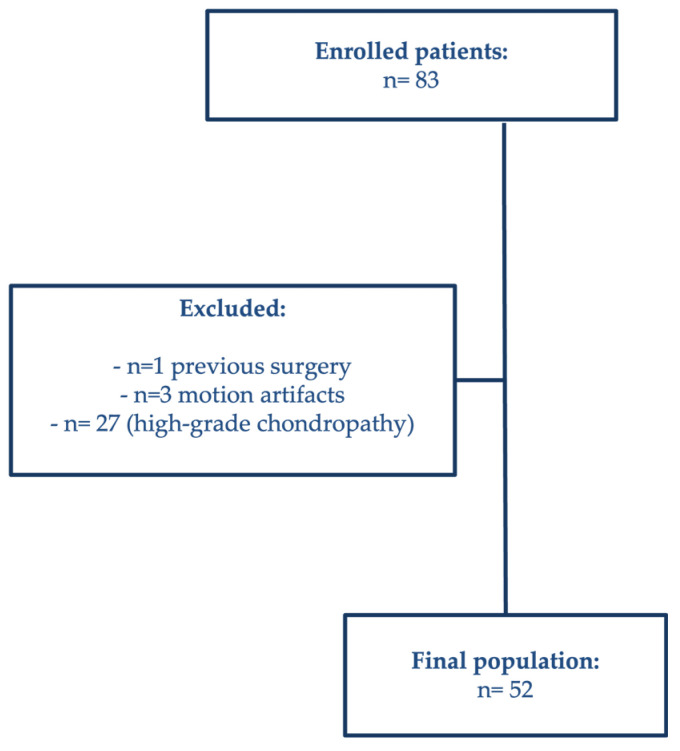
Flowchart of patient enrolment showing inclusion and exclusion criteria.

Patients with previous surgery (*n* = 1) and images affected by severe motion artifacts (*n* = 3) were excluded. The remaining 27 patients were excluded because preoperative imaging demonstrated high-grade chondropathy (grade III–IV) (Figure 1).

### 3.2. MRI Scanner and Acquisition Protocol

For each subject, two sagittal acquisitions were obtained using different flip angles (30° and 60°). From these datasets, T2 relaxometric maps were automatically generated through the 3D SHARC sequence. Each patient dataset consisted of a total of 156 images. The mean total acquisition time was 8.33 ± 14.3 min.

### 3.3. Image Analysis

**Figure 2:**
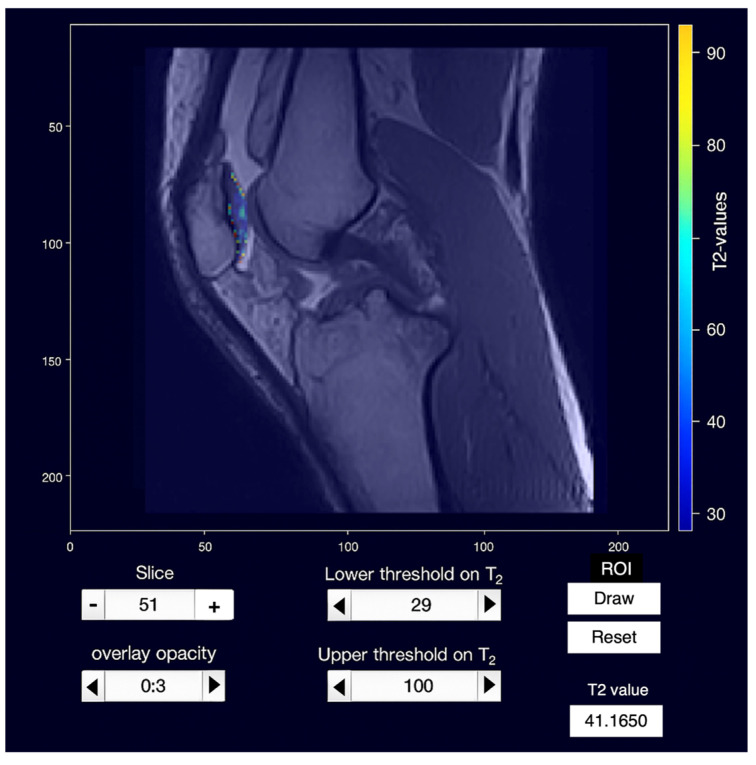
Experimental software interface for T2 mapping analysis. A free-hand region of interest (ROI) is manually drawn over the cartilage, and a colorimetric map is generated, with different colors representing various T2 relaxation times. The mean T2 relaxation time within the ROI is displayed at the bottom of the interface.

**Figure 3:**
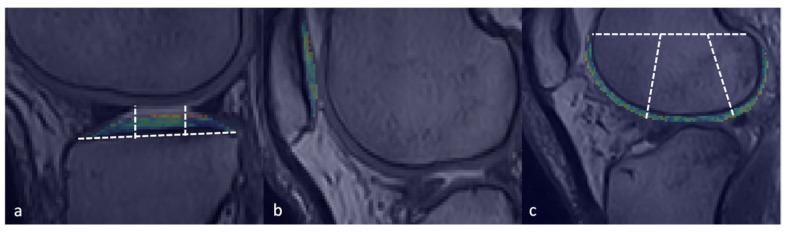
Image analysis according to the Whole-Organ Magnetic Resonance Imaging Score (WORMS) classification. Representative examples of manually drawn regions of interest (ROIs) for T2 relaxation time measurements are shown with dashed lines for the (**a**) tibial plateau, (**b**) patella, and (**c**) femoral condyle.

A total of 728 measurements were performed with the dedicated software (Figure 2 and Figure 3).

**Figure 4:**
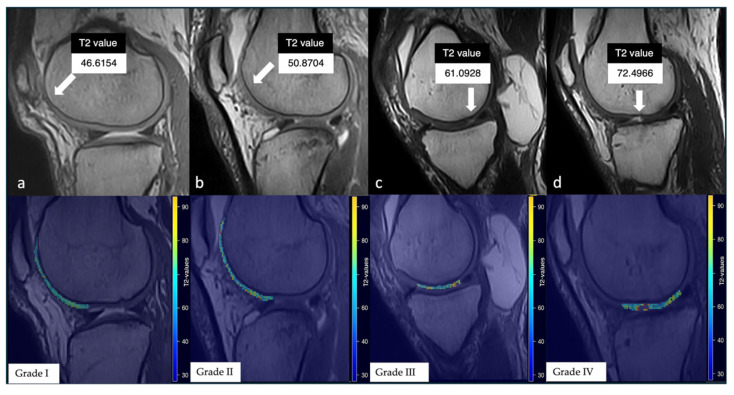
Representative examples close to the median T2 values for each chondropathy grade on both the 3D SHARC sequence (top row) and the corresponding T2 relaxation time maps (bottom row). White arrows highlight the analyzed cartilage regions. Progressive increases in T2 values are observed with increasing cartilage damage severity: (**a**) grade I, (**b**) grade II, (**c**) grade III, and (**d**) grade IV.

All different grades of chondropathy were compared among them (Figure 4).

T2 mapping differentiated healthy cartilage (grade 0) from pathological cartilage (grades I–II), with an optimal cut-off of 45 ms (AUC ≈ 0.70, 0.714 for Reader 1 and 0.709 for Reader 2). Agreement with arthroscopy was good (weighted κ = 0.731). Intra-reader reproducibility was excellent (ICC = 0.998 for both readers), and inter-reader agreement was good (ICC = 0.753). Interquartile T2 ranges were 36–47 ms for grade 0, 40–50 ms for grade I, and 44–52 ms for grade II, with marked overlap between grades I and II (Table 2 and Table 3).

### 3.4. Arthroscopy

At arthroscopic evaluation, most cartilage sites were classified as grade 0 (*n* = 434, 61%), indicating preserved surface integrity and normal cartilage appearance. Grade I lesions (*n* = 127, 18%) showed superficial fibrillation or softening without visible surface disruption, while grade II lesions (*n* = 148, 21%) presented partial-thickness defects involving less than 50% of the cartilage depth. The majority of degenerative changes were observed at the femoral condyles (*n* = 276, 59%), particularly in the central and posterior regions, whereas fewer abnormalities were detected at the tibial plateaus (*n* = 142, 30%) and patellar surfaces (*n* = 41, 9%). Minimal involvement was observed in other joint regions (*n* = 10, 2%) (Table 4).

## 4. Discussion

In this prospective diagnostic accuracy study, T2 mapping performed on a low-field MRI system demonstrated good agreement with arthroscopic findings in detecting early chondral changes, with high reproducibility and a fair discriminative ability between normal and early pathological cartilage.

The progressive increase in T2 relaxation time observed with cartilage degeneration can be explained by structural and biochemical alterations within the extracellular matrix. The normal hyaline cartilage consists of type II collagen fibers, proteoglycans, and water, with a highly organized collagen network that restricts water mobility. As degeneration progresses, disruption and disorganization of the collagen framework occur, leading to increased mobility of water protons and longer T2 relaxation times. Moreover, loss of proteoglycans reduces the fixed charge density and decreases the proportion of bound water, further enhancing free water content. These combined effects result in prolonged T2 values reflecting both increased hydration and collagen disarray [14,15].

For the purpose of this study, we focused exclusively on early stages of chondropathy. High-grade cartilage lesions (Outerbridge grade III–IV) were not included, as they can be reliably identified through conventional morphological MRI sequences or even standard radiographs. Indeed, a systematic review published in 2015 [16] demonstrated that the sensitivity of conventional radiography for detecting mild osteoarthritis (Outerbridge grade II) is low, ranging from as little as 3% to 75% depending on the compartment and projection used. In contrast, sensitivity markedly increases for advanced disease, reaching values as high as 86% with the 45° posteroanterior (PA) view and up to 95% with Kellgren–Lawrence criteria in the medial compartment. These findings underline the limited utility of radiographs for early chondral changes and support the use of more advanced techniques—such as quantitative mapping, especially in early stages of OA—to improve diagnostic accuracy in the initial phases of cartilage degeneration.

In addition, V. M. Mattila et al. [17] reported a sensitivity of up to 100% for detecting grade III–IV chondral lesions using 1.0 T MRI, further confirming that conventional imaging is only highly reliable in advanced disease stages. In our study, we aimed to overcome this limitation by translating T2 mapping results into quantitative relaxometry thresholds capable of distinguishing normal cartilage from early degenerative changes. Based on arthroscopic correlation, T2 mapping enabled differentiation between healthy cartilage (grade 0) and pathological cartilage (grades I–II) with an optimal cut-off value of 45 ms (AUC ≈ 0.70 for both readers). Interquartile T2 ranges were 36–47 ms for grade 0, 40–50 ms for grade I, and 44–52 ms for grade II. Despite the marked overlap between grades I and II, this quantitative approach allowed for reliable identification of early cartilage alterations that were typically below the detection threshold of conventional MRI or radiography.

We also found increased T2 relaxation time values when comparing normal cartilage with grade I and grade II chondropathy confirmed at arthroscopic evaluation. The observation that T2 relaxation time progressively increases with the severity of cartilage damage is consistent with findings from previous studies at 1.5 T and 3.0 T, which reported a significant positive correlation between T2 values and cartilage degeneration grade. For example, Cao et al. (2023) [18] demonstrated correlation coefficients as high as r = 0.773 (superior band), r = 0.750 (middle band), and r = 0.723 (inferior band), while Apprich et al. [19] reported a significant increase in global T2 values from approximately 38.9 ms in normal cartilage to 41.2 ms in grade I and 47.7 ms in grade II lesions (*p* = 0.041), with the strongest correlation observed in the superficial layer (r = 0.620, *p* < 0.0001). This progressive increase reflects early biochemical changes, including elevated water content, proteoglycan depletion, and disruption of the collagen network. Moreover, Apprich et al. [19] reported that three patients with morphologically normal cartilage exhibited T2 values exceeding 45 ms, higher than the mean observed for grade I lesions (41.2 ms), suggesting that quantitative T2 mapping may detect preclinical matrix alterations that remain invisible on morphological MRI. However, in our cohort, the diagnostic accuracy of T2 mapping for differentiating normal cartilage from early chondropathy was moderate, with an AUC of approximately 0.70 for both readers. This relatively modest discriminative performance likely reflected the substantial overlap of T2 values between grade I and grade II lesions (interquartile ranges: 40–50 ms for grade I and 44–52 ms for grade II), which is a well-recognized limitation of quantitative mapping in the early stages of cartilage degeneration. Similar findings were reported by Cao et al. [18], who observed that mean T2 values rose from approximately 36.5 ± 3.1 ms in normal cartilage to 44.7 ± 4.2 ms in mild degeneration and 51.2 ± 5.6 ms in severe degeneration (*p* < 0.01). Despite these limitations, the significant increase in T2 relaxation time observed even in grade I lesions underlines the clinical utility of this technique in detecting early cartilage matrix alterations before gross morphological changes become visible on conventional MRI. These findings—previously demonstrated mainly at high magnetic field strengths—appear to also be reproducible on low-field MRI (<0.5 T), supporting the potential of low-field T2 mapping as a cost-effective and widely accessible tool for the early detection of cartilage degeneration in routine clinical practice.

S.T. Soellner et al. [20] conducted a study similar to ours, comparing T2 relaxation times of knee cartilage with intraoperative arthroscopic findings to establish quantitative reference values for differentiating healthy from degenerated cartilage. They examined 21 patients using a T2 mapping sequence at 3.0 T MRI, and cartilage quality was subsequently assessed arthroscopically according to the International Cartilage Repair Society (ICRS) classification. The articular cartilage was divided into six regions of interest, and a progressive increase in T2 values was observed with higher ICRS grades. Specifically, mean T2 relaxation times were 38.97 ± 6.78 ms for grade 0 (normal cartilage), 55.21 ± 7.20 ms for grade I, 73.06 ± 11.55 ms for grade II, and 97.16 ± 14.88 ms for grade III. A statistically significant and strong positive correlation was found between T2 relaxation times and cartilage degeneration (ρ = 0.940, *p* < 0.0001). Furthermore, ROC curve analysis identified a cut-off value of 48.7 ms for distinguishing normal cartilage from early degeneration, with an AUC of 0.963, sensitivity of 95.1%, and specificity of 88.9%, confirming the high diagnostic accuracy of T2 mapping in this context. Although their results were obtained using a 3.0 T MRI scanner, our findings at 0.31 T demonstrated a similar trend of progressive T2 increase with cartilage degeneration, confirming that this quantitative parameter reliably reflects early biochemical and structural changes even at lower magnetic field strengths. This suggests that low-field MRI, despite its intrinsic limitations in signal-to-noise ratio and spatial resolution, can still provide clinically meaningful information for the detection and characterization of early chondral pathology when combined with advanced mapping techniques.

Richard Kijowski et al. [6] evaluated 150 patients with 3.0 T MRI and demonstrated that the addition of a T2 mapping sequence markedly increased the sensitivity for detecting early cartilage degeneration from 74.6% to 88.9% overall, and from 4.2% to 62% for the earliest grade IA lesions. They also reported characteristic T2 relaxation values of 20–30 ms in the deep layer and 40–50 ms in the superficial layer of normal cartilage, reflecting zonal collagen organization. These results highlight the value of quantitative mapping in revealing early biochemical changes that remain undetectable on conventional sequences. Consistent with these findings, our study shows that even at 0.31 T, T2 mapping follows the same trend, suggesting that this technique can also substantially improve diagnostic accuracy for early chondral lesions in low-field clinical settings.

Early detection of chondral damage has significant clinical implications, as it can influence therapeutic decision-making and allow for timely initiation of conservative treatments before irreversible structural changes occur. Recent studies have shown that interventions such as platelet-rich plasma (PRP) or intermediate-weight hyaluronic acid (HA) injections are associated with significant pain reduction and functional improvement in patients with low-grade chondropathy [21,22]. Moreover, Bellisari et al. [4] demonstrated that T2 mapping not only provides valuable information on cartilage water content and matrix integrity but also serves as a useful biomarker to monitor treatment response, showing significant reductions in T2 relaxation times after PRP injections compared to baseline. These findings support the notion that early quantitative MRI assessment could help identify patients most likely to benefit from non-surgical interventions, thereby optimizing clinical outcomes.

## 5. Conclusions

T2 mapping performed with a low-field MRI system demonstrated good agreement with arthroscopy and high reproducibility in detecting early cartilage degeneration. Although its discriminative performance was moderate, quantitative assessment of T2 relaxation times may provide indirect information on early matrix changes that remain undetectable with conventional imaging. These findings suggest the potential role of low-field T2 mapping as a cost-effective and accessible technique for the early evaluation of chondral pathology. Future head-to-head studies comparing 0.31 T and 3.0 T T2 mapping are encouraged to better define the diagnostic trade-offs and clinical applicability of low-field imaging.

## Figures and Tables

**Table 1:** Characteristics of patients enrolled.

Total *	*n* = 52	
Sex *	Male	*n* = 32
	Female	*n* = 20
*Age (Years) ^†^*		48.05 ± 17.18 (18–71)
*BMI (Kg/m^2^) ^†^*		25.01 ± 3.36

* Data are frequencies. ^†^ Data are mean ± standard deviation. BMI: body mass index.

**Table 2:** Agreement and reproducibility metrics for T2 mapping showing moderate discriminative accuracy (AUC ≈ 0.70, 0.714 for Reader 1 and 0.709 for Reader 2), good agreement with arthroscopy (κ = 0.731), excellent intra-reader reproducibility (ICC = 0.998), and substantial inter-reader agreement (ICC = 0.752).

**Parameter**	**Reader 1**	**Reader 2**	**Overall/Between Readers**
AUC (ROC)	0.714	0.709	-
Weighted Cohen’s Κ	-	-	0.731
ICC (Intra-Reader)	0.998	0.998	-
ICC (Inter-Reader)	-	-	0.752

**Table 3:** T2 values showed a progressive increase from grade 0 to grade II. Median T2 values were approximately 42 ms for grade 0, 45 ms for grade I, and 48 ms for grade II. Despite this trend, a substantial overlap was observed between grades I and II, preventing a reliable distinction between the two.

**Cartilage Grade**	**Interquartile Range (ms)**	**Median (ms)**
Grade 0	36–47	~42
Grade I	40–50	~45
Grade II	44–52	~48

**Table 4:** Distribution of cartilage grades (0–II) across anatomical subregions, showing a predominance of grade 0 findings at all femorotibial and patellar sites, with grade I and II lesions more frequently observed at the femoral condyles and tibial plateaus.

**Anatomical Site**		**Grade 0 (N, %)**	**Grade I (N, %)**	**Grade II (N, %)**	**Total**
Medial Femoral Condyle	Anterior	27 (54%)	6 (12%)	17 (34%)	50
	Central	29 (59%)	7 (14%)	13 (27%)	49
	Posterior	31 (61%)	10 (20%)	10 (20%)	51
Lateral Femoral Condyle	Anterior	32 (64%)	9 (18%)	9 (18%)	50
	Central	30 (61%)	5 (10%)	14 (29%)	49
	Posterior	33 (65%)	5 (10%)	13 (25%)	51
Medial Tibial Plateau	Anterior	29 (58%)	11 (22%)	10 (20%)	50
	Central	30 (60%)	9 (18%)	11 (22%)	50
	Posterior	28 (56%)	13 (26%)	9 (18%)	50
Lateral Tibial Plateau	Anterior	32 (62%)	11 (21%)	9 (17%)	52
	Central	32 (63%)	11 (22%)	8 (16%)	51
	Posterior	30 (58%)	13 (25%)	9 (17%)	52
Patella	Medial	36 (69%)	7 (13%)	9 (17%)	52
	Lateral	35 (67%)	10 (19%)	7 (13%)	52
Total		434 (61%)	127 (18%)	148 (21%)	709

## Data Availability

The raw data supporting the conclusions of this article will be made available by the authors on request.

## Abbreviations

The following abbreviations are used in this manuscript:

OA	Osteoarthritis
PG	Proteoglycan
ROIs	Region of interest
MRI	Magnetic resonance imaging
T2W	T2-weighted
T1W	T1-weighted
WORMS	Whole-Organ Magnetic Resonance Imaging Score
ACL	Anterior cruciate ligament
ICRS	International Cartilage Repair Society
CT	Computed tomography
BME	Bone marrow edema
WOMAC	Western Ontario and McMaster Universities Osteoarthritis Index
KOOS	Knee injury and Osteoarthritis outcome score
